# A Validated WISC-V Short-Form to Estimate Intellectual Functioning in Very Preterm Children at Early School Age

**DOI:** 10.3389/fpsyg.2021.789124

**Published:** 2021-12-17

**Authors:** Andone Sistiaga, Joana Garmendia, Jone Aliri, Itxaso Marti, Garazi Labayru

**Affiliations:** ^1^Department of Clinical and Health Psychology and Research Methodology, Psychology Faculty, University of the Basque Country (UPV/EHU), Donostia-San Sebastián, Spain; ^2^Neuroscience Area, Biodonostia Health Research Institute, Donostia-San Sebastián, Spain; ^3^Paediatric Department, Donostia University Hospital, Donostia-San Sebastián, Spain; ^4^Paediatric Department, Faculty of Medicine, University of the Basque Country (UPV/EHU), Donostia-San Sebastián, Spain

**Keywords:** very preterm, neurodevelopment, IQ, WISC-V, short-form, validation

## Abstract

Very preterm children (gestational age < 32 weeks) frequently show neurodevelopmental difficulties (Inattention/dysexecutiveness) throughout their life-stages. A scarcity of resources, along with this population’s cognitive vulnerability, makes the neuropsychological evaluation of these children both complicated and time-consuming. This study aimed to develop a specific and valid Wechsler Intelligence Scale for Children-Fifth Edition (WISC-V) short-form to estimate intellectual functioning in this population. Eighty-four very preterm children (39 female; mean age = 6.50; *SD:* 0.06) were assessed with the WISC-V. Short-forms were developed following two independent strategies: a) multiple linear regressions for each index; b) correlational analyses between scores on all administered subtests and Full-Scale IQ. Validity of short-forms was analyzed. A short-form (Vocabulary, Matrix Reasoning, Picture Span, and Symbol Search) that satisfied 2/3 validation criteria was proposed. This validated short-form could facilitate the identification of cognitive difficulties in very preterm children, so that they could benefit from early care and support services, avoiding long assessment procedures.

## Introduction

Preterm birth, that is, delivery before 37 weeks of gestation, is a major obstetric and global health problem, and it is the second most frequent cause of death in children under 5 years old worldwide (Blencowe et al., 2012). Those infants who do survive suffer long-term morbidity, including neurological and developmental disabilities, compared to infants born full-term (Allotey et al., 2018).

Major neurodevelopmental morbidities are usually detected during the first 2 years of life. However, the effect of prematurity on various neurodevelopmental domains persists up to secondary school age and beyond and could even increase as academic expectations rise (Pritchard et al., 2014). The early years of primary school are critical since children are asked to meet increasing academic demands and any likely difficulties could begin to impact school performance. For that reason, it is important to design neuropsychological assessment protocols for early school-age preterm children, to promptly identify and trace the potential neurodevelopmental difficulties that might emerge in these school beginners (Jansen et al., 2021).

Risks of adverse outcomes rise significantly with decreasing gestation time, which has lead to a focus on research and clinical assistance in very preterm children (i.e., children born before 32 weeks of gestational age), considered a vulnerable group within preterms (Delobel-Ayoub et al., 2009). This group is known to have cognitive deficits affecting overall intelligence, estimated to be on average 0.7 SD below their peers (Anderson, 2014), along with problems in other cognitive domains such as attention and executive functions (Brydges et al., 2018). Thus, a neuropsychological assessment that includes estimating general intellectual functioning is crucial to depict the global cognitive performance and its potential long-term evolution in very preterm children. However, IQ estimation takes long and is frequently insufficient. Assessment procedures incorporating specific cognitive domains are usually required, but these are generally time-consuming, and the health care system may not always have the resources to respond to this demand. For this reason, shortened assessment protocols are preferable, particularly when considering the inattentive/dysexecutive profile frequently shown by very preterm children, which might cause biased results due to the effect of fatigue and distractibility effects (Franz et al., 2018).

The Wechsler Intelligence Scale for Children (WISC) is the most frequently used assessment tool to estimate intellectual functioning in children and adolescents. Although several works have been conducted to develop WISC short-forms, most are proposed for the general population and may not be valid for specific clinical populations. For that reason, the scientific literature recommends validating short-forms in specific populations (Van Ool et al., 2018), as has been previously done by other authors in various neurological conditions such as traumatic brain injury, epilepsy, and neurodevelopmental disorders, among others (Donders et al., 2013; Hrabok et al., 2014; Lace et al., 2020).

At present, there is no specific WISC short-form for estimating general intellectual functioning in very preterm children. However, brief tools can enable the prompt identification of neurodevelopmental difficulties in very preterm children so that further cognitive assessment (including critical cognitive domains in this population) can be implemented in a timely manner. Therefore, this study aimed to develop a psychometrically appropriate WISC-V short-form to estimate Full-Scale IQ (FSIQ) in this vulnerable clinical population at early school age.

## Materials and Methods

### Participants

Participants in this study are part of the longitudinal preterm cohort at the reference Neonatology Unit in the current geographical area (children born between 2010 and 2013, in Donostia University Hospital). Inclusion criteria for this study included being born before 32 weeks of gestation and having no other neurological or psychiatric disorders at that time. Eighty-four (39 female, 46.4%) out of 101 invited cohort participated in the study, and 17 children were excluded (10 refused to participate, four were unreachable, two were unavailable, and one child did not collaborate). The mean age of the participants at the neuropsychological assessment was 6.5 years (*SD:* 0.06). A description of the participants is presented in Table 1.

**TABLE 1:** Demographic and clinical data.

	*M*	*SD*	Min	Max
Age (years)	6.50	0.06	6.00	6.57
Gestational age (months)	29.31	1.94	24.86	31.86
FSIQ	90.98	14.07	47	130
VCI	90.95	16.20	0	124
VSI	90.95	16.20	57	126
FRI	95.13	11.86	58	123
WMI	90.04	15.55	55	142
PSI	94.48	16.54	45	141
Block design	9.21	2.88	2	15
Similarities	7.65	2.79	0	15
Matrix reasoning	8.93	2.58	3	15
Digit span	7.76	2.77	2	16
Coding	8.93	3.14	1	18
Vocabulary	9.19	2.80	0	15
Figure weights	9.42	2.19	1	13
Visual puzzles	9.71	2.73	3	15
Picture span	8.80	3.38	1	19
Symbol search	9.10	3.13	1	16

*M, mean; SD, standard deviation; FSIQ, Full-Scale IQ; VCI, Verbal Comprehension Index; VSI, Visual-Spatial Index; FRI, Fluid Reasoning Index; WMI, Working Memory Index; PSI, Processing Speed Index; FSIQ score range, 40–160 (M = 100; SD = 15); Indexes score range, 45–155 (M = 100; SD = 15); Subtests score range, 1–19 (M = 10; SD = 3).*

The parents of all participants signed the informed consent. The study was approved by the Ethics Committee for Clinical Investigation of the health area of Gipuzkoa (ASB-PLP-2017-01).

### Intellectual Functioning Assessment

The assessment, which consisted of administering the Spanish version of the WISC-V (Wechsler, 2015) was conducted between 2017 and 2019 by two experienced neuropsychologists, just at the time the children of the cohort were 6.5 years old.

The WISC-V includes 15 subtests: seven core subtests and eight supplementary subtests. Structurally, this instrument is organized into five indexes: Verbal Comprehension, Visual-Spatial, Fluid Reasoning, Working Memory, and Processing Speed. Based on the standardized scores of the seven core subtests (Block Design, Similarities, Matrix reasoning, Digit Span, Coding, Vocabulary and Figure Weights), a Full-Scale Intelligence Quotient (FSIQ) is calculated (estimated administration time for 6 years old children: 40 min).

**FIGURE 1:**
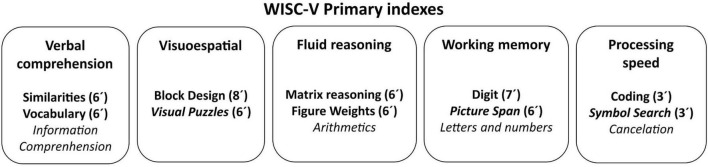
WISC-V Primary indexes and their subtests. All WISC-V subtests are included in the figure. The main subtests needed for calculating the indexes and the estimated administration time (in minutes) for each are displayed in bold.

In this study, all the subtests required for calculating the indexes were administered (see Figure 1 and Supplementary Table 1 for detailed information). Raw scores of the subtests were converted into scaled scores, following Spanish normative data according to age group.

One of the most notable updates of the WISC-V is the division of the Perceptual Reasoning index (WISC-IV) into two different indexes: Fluid Reasoning and Visual-Spatial indexes.

### Statistical Analysis

#### Development of the Short-Forms

The data were analyzed using the SPSS statistical package (Version 24). Two independent strategies were followed to obtain WISC-V short-forms. The first strategy aimed to maintain the factorial structure of the scale. To this end, independent multiple linear regression analyses for each of the five indexes were conducted (after checking the corresponding assumptions), to select the subtest with the greatest predictive capacity for each index. The second strategy focused on selecting subtests closely related to the FSIQ. For this purpose, Spearman’s correlation analyses were conducted between all administered WISC-V subtests and the FSIQ. Only subtests with a high correlation (rho ≥ 0.7) were selected, indicating a very strong association (Davis, 1971).

Moreover, to provide highly reliable short-forms, the internal consistency reliabilities were directly estimated from the study sample using Spearman-Brown formula, with the exception of time limited subtests (Symbol Search and Coding) for which test-retest reliabilities from the standardization sample were used (Wechsler, 2015; Supplementary Table 2). These coefficients were based on the sample of this study, and only subtests with *r_*xx*_* ≥ 0.8 were selected.

IQ estimations of the resulting short-forms were obtained using the formula described by Tellegen and Briggs (1967), where subtests inter-correlations and internal consistency reliabilities were directly estimated from the preterm sample when possible. Consequently, a multiple linear regression was conducted for each short-form, with the subtests comprising the short-form as predictors and the FSIQ as the criterion variable. In all cases, only subtests that reached statistical significance were included.

#### Validation of the Short-Forms

The developed short-forms were interpreted following the criteria established by Donders and Axelrod (2002): (a) reliability coefficient ≥ 0.90 of the short-form; (b) a corrected correlation ≥ 0.82 between FSIQ and short-form estimated IQ; (c) a proportion ≥ 0.81 of short-form estimates that fell within the 90% confidence interval (CI) of the FSIQ (i.e., within ± 7 FSIQ points). The reliability coefficient of the short-forms and the corrected correlation between FSIQ and the short-form estimated IQ were calculated using the above mentioned formula (Tellegen and Briggs, 1967).

## Results

### Development of the Short-Forms

From each statistical analysis strategy, one short-form proposal was obtained: proposal A (from the first strategy) and proposal B (from the second strategy).

For proposal A, the subtests with the highest standardized β coefficient in each regression analysis were as follows: Vocabulary subtest (standardized β coefficient: 0.57) in the Verbal Comprehension Index, Block Design subtest (standardized β coefficient: 0.57) in the Visual-Spatial Index, Matrix Reasoning (standardized β coefficient: 0.64) in the Fluid Reasoning Index, Picture Span subtest (standardized β coefficient: 0.64) in the Working Memory Index, and Symbol Search subtest (standardized β coefficient: 0.55) in the Processing Speed Index (see Supplementary Table 3 for assumptions and Supplementary Table 4 for more detailed information of the results of those linear regression analyses). In addition, the Block Design subtest was removed since its reliability coefficient (*r_*xx*_* = 0.75) failed to reach *r_*xx*_* ≥ 0.8.

The linear regression model executed with the combination of Vocabulary, Matrix Reasoning, Picture Span, and Symbol Search scores explained 84% of the variance of the FSIQ, and all selected subtests were statistically significant predictors of the FSIQ (*p* < 0.05) (see Table 2).

**TABLE 2:** Linear regressions between short-forms and FSIQ.

Variable	*B*	*SE*	*T*	*p*	95% CI
**Proposal A**					
(Constant)	37.89	2.61	14.49	0.000	[32.68–43.09]
Vocabulary	1.92	0.25	7.55	0.000	[1.41–2.43]
Matrix reasoning	2.17	0.30	7.29	0.000	[1.58–2.76]
Picture span	0.50	0.22	2.33	0.022	[0.07–0.93]
Symbol search	1.28	0.26	4.99	0.000	[0.77–1.79]
**Proposal B**					
(Constant)	43.13	2.38	18.13	0.000	[38.40–47.87]
Matrix reasoning	2.36	0.29	8.23	0.000	[1.79–2.93]
Digit span	1.27	0.30	4.26	0.000	[0.68–1.86]
Coding	1.89	0.25	7.43	0.000	[1.39–2.40]

*SE, Standard Error.*

For proposal B, the results from the Spearman’s correlation analysis are displayed in Table 3. Again, the subtests Matrix Reasoning, but also Digit Span, and Coding yielded strong correlations (rho > 0.7), and their reliability coefficient reached *r_*xx*_* ≥ 0.8.

**TABLE 3:** Spearman’s Correlations between FSIQ and the subtests of the scales.

	BD	SI	MR	DS	CD	VC	FW	VP	PS	SS
*Rho*	0.66**	0.65**	0.76**	0.74**	0.77**	0.67**	0.53**	0.63**	0.52**	0.69**

BD, Block Design; SI, Similarities; MR, Matrix Reasoning; DS, Digit Span; CD, Coding; VC, Vocabulary; FW, Figure Weights; VP, Visual Puzzles; PS, Picture Span; SS, Symbol Search. The shaded areas in the table illustrate the subtests with rho ≥ 0.7. **p < 0.01 (two-tailed).

The regression model composed of Matrix Reasoning, Digit Span, and Coding subtests explained 84% of the FSIQ variance. Moreover, all the subtests were statistically significant predictors of the FSIQ (*p* < 0.05), as shown in Table 2.

### Validation of the Short-Forms

For proposal A, the results of the validation criteria were (a) the reliability coefficient was 0.95; (b) the corrected correlation between FSIQ and the estimated IQ was 0.90, and (c) the proportion of estimations within the CI 90% was 0.67. This proposal accomplished the first two validation criteria (a and b). In addition the mean difference between FSIQ and the estimated IQ of Proposal A was –1.48 (SD = 7.80; min = –21.78 and max = 17.34).

For proposal B, the results of the validation criteria were (a) the reliability coefficient was 0.88; (b) the corrected correlation between FSIQ and the estimated IQ was 0.72, and (c) the proportion of estimations within the 90% CI was 0.79. This proposal did not accomplished any of the validation criteria. In addition the mean difference between FSIQ and the estimated IQ of Proposal B was –0.26 (*SD* = 5.80; min = –13.00 and max = 12.00).

## Discussion

Most of the neuropsychological research studies on very preterm children have administered the full WISC scale, even though this considerably reduces the time for assessing other clinically concerning specific cognitive domains. However, population-specific and validated short-forms can contribute toward easing and simplifying intellectual performance assessment. Besides, this could provide better psychometric properties than general population short-forms, given that a specific short-form is more suitable for a clinical population. To date, only two studies have developed IQ estimation short-forms for the WISC-V (Aubry and Bourdin, 2018; Lace et al., 2020), neither of which targeted preterm children. Encouraged by this, the present study provides, for the first time, a specific and validated WISC-V short-form for estimating intellectual functioning in very preterm children.

With regard to validation of the short-form, the results from this study are interpreted following the most recent validation criteria (Donders and Axelrod, 2002), and data regarding validity and reliability in very preterm population are provided.

The results from this study provided two short-forms: the first was methodologically based on the factorial structure of the test and resulted in a four subtest short-form (Vocabulary, Matrix Reasoning, Picture Span, and Symbol Search), and satisfied two out of three of the validation criteria. The second, completely data-driven, was composed of three subtests (Matrix Reasoning, Digit Span, and Coding) and did not fulfill any of the validation criteria.

Although the second strategy yielded a shorter form, its psychometric properties were insufficient to reach the validity threshold. However, the first proposal met the reliability and corrected correlation criteria. Although it did not fulfill the last criterion (proportion of estimations within 90% CI), the mean discrepancy between the FSIQ and the IQ obtained from the short-form was generally small, and 66.67% of the estimated IQs were within the 90% CI of the FSIQ. This last criterion is proposed for the clinical application of short-forms and it is important to note that several studies, including Donders and Axelrod (2002), have reported difficulties in satisfying this criterion (Chen and Hua, 2020).

In light of the obtained results, we recommend the use of the four subtests short-form (proposal A). Although the Tellegen and Briggs (1967) formula was used for calculating IQ values of the short-form, prorating can be used by clinicians to simplify IQ acquisition, given the high correlation between the IQ estimations obtained by both methods in this study (r: 0.99). In this case, prorated IQ is obtained by the sum of the scaled scores of the short-form subtests and multiplied by 7/4 (number of subtests required for calculating FSIQ/number of subtests of the short-form). The obtained score needs to be converted to FSIQ according to the WISC-V normative data.

This short-form provides an estimation of the general intellectual functioning in half of the time taken to complete the full scale (20′ as opposed to the 40′ needed for FSIQ), while maintaining the following four-factor structure: Verbal Comprehension, Visual-Spatial/Fluid Reasoning, Working Memory, and Processing Speed. Although WISC-V is structured into five indexes, previous work suggests that the WISC-V may better fit a four-factor model, combining Visual-Spatial and Fluid Reasoning indexes (Dombrowski et al., 2018; Canivez et al., 2020).

Two of the subtests included in the short-form (Vocabulary and Matrix Reasoning) are included in the Wechsler Abbreviated Scale of Intelligence-II (WASI-II) (Wechsler, 2011) and other brief intelligence tools such as the K-BIT (Kaufman and Kaufman, 1990). As these subtests cover the verbal domain and aspects of fluid reasoning, these have been more frequently used for short-form combinations. However, the assessment of executive functions such as working memory and processing speed is particularly compromised in children with neurodevelopmental vulnerability, as in the case of very preterm children (Houdt et al., 2019; Schnider et al., 2020), and is not included in these abbreviated instruments. According to some authors, this could lead to an IQ overestimation of very preterm children (Nosarti et al., 2007).

In contrast, our proposal covers these domains with Picture Span and Symbol Search subtests. Furthermore, in line with some authors’ recommendation to develop and validate specific short-forms for the clinical population (Lace et al., 2020), the tetrad proposed in this study takes into account the particular developmental trajectory of this population.

Aside from the intrinsic limitations associated with the use of short-forms (these tests are not suitable for diagnostic or specific classification purposes; Van Ool et al., 2018), the main constraint of the present study is the small sample size. This limitation is due to the fact that we included only the most vulnerable preterm children (the very preterms) which limits the generalizability of the results to a broader population of preterm children. Further research may seek to replicate and extend these findings in larger samples of preterm children, including moderate to late preterms.

## Conclusion

In conclusion, a specific and validated WISC-V short-form was developed for very preterm children that might provide better reliability and fit better with the cognitive profile of this clinical population than general population short-forms. This work provides a brief cognitive tool that can facilitate the neuropsychological assessment and follow-up of very preterm children to identify special needs in this population, so that they could benefit from optimal care or early intervention programs. Moreover, this work could contribute further to the systematization of neuropsychological assessment in very preterm children, favoring the comparison between studies.

## Data Availability Statement

The raw data supporting the conclusions of this article will be made available by the authors, without undue reservation.

## Ethics Statement

The studies involving human participants were reviewed and approved by Ethics Committee for Clinical Investigation of the health area of Gipuzkoa (ASB-PLP-2017-01). Written informed consent to participate in this study was provided by the participants’ legal guardian/next of kin.

## Author Contributions

AS and JG contributed to conceptualization, methodology, writing—original draft, and editing. JA contributed to methodology, formal analysis, writing—review, and editing. IM contributed to conceptualization, writing—review, and editing. GL contributed to conceptualization, methodology, investigation, data curation, writing—review, and editing. All authors contributed to the article and approved the final manuscript.

## Conflict of Interest

The authors declare that the research was conducted in the absence of any commercial or financial relationships that could be construed as a potential conflict of interest.

## Publisher’s Note

All claims expressed in this article are solely those of the authors and do not necessarily represent those of their affiliated organizations, or those of the publisher, the editors and the reviewers. Any product that may be evaluated in this article, or claim that may be made by its manufacturer, is not guaranteed or endorsed by the publisher.
