# Supplementary material for: A Validated WISC-V Short-Form to Estimate Intellectual Functioning in Very Preterm Children at Early School Age
Source: Front Psychol. 2021 Dec 17;12:789124. doi: 10.3389/fpsyg.2021.789124 (PMC8718391; doi:10.3389/fpsyg.2021.789124)
Supplement: Supplementary file 1 [file Data_Sheet_1.docx]

**Supplementary Data**

**Supplementary Table 1**

*Description of the administered WISC-V subtests*

| Subtest | Description | Index | Category |
| --- | --- | --- | --- |
| Similarities (SI) | -This requires the participant to describe a similarity between two words that represent a common object or concept.  -This measures the ability to express the relationships between two concepts: associative thinking and the capacity for verbal abstraction. | VCI | Principal for FSIQ |
| Vocabulary (VC) | -This requires the participant to name depicted objects (naming) and/or to define words that are read aloud by the examiner.  -This reflects the level of education, the learning capacity, the formation of verbal concepts and the verbal and semantic richness of the environment in which the evaluated person operates. | VCI | Principal for FSIQ |
| Block Design (BD) | -This requires the participant to build drawings with red and white colored cubes of increasing complexity.  -This evaluates the ability to analyze and visually synthesize, and to reproduce abstract geometric drawings. | VSI | Principal for FSIQ |
| Visual Puzzles (VP) | -This involves choosing the three pieces that complete a puzzle, which is presented as a completed model.  -It measures the ability to analyze and synthesize abstract visual stimuli, establish relationships between parts, and non-verbal reasoning. | VSI | Principal for VSI |
| Matrix Reasoning (MR) | -In this test the participant must choose the drawing that completes an incomplete series.  -It measures abstract reasoning and the ability to process visual information. | FRI | Principal for FSIQ |
| Figure Weights (FW) | -Balances with two dishes are presented and in one of them there are one or more weights. The task is to select the weight, or weights, that balance the scale dishes.  -This measures the ability to compare, establish analogues and quantitative reasoning. | FRI | Principal for FSIQ |
| Digit Span (DS) | -This consists of three tasks: direct digits (repeating a series of digits, which are presented orally, in the same order as presented); reverse digits (repeating a series of digits in reverse order as presented); and digits in increasing order (repeating numbers read by the examiner from lowest to highest).  -It evaluates attention and resistance to distraction, immediate auditory memory, and working memory. | WMI | Principal for FSIQ |
| Picture Span (PS) | -This involves observing a series of drawings for a certain time, and then selecting them from among other drawings, marking them in the same order as they were presented.  -It evaluates visual memory and working memory capacity. | WMI | Principal for WMI |
| Coding (CD) | -In this task, digits are shown, which are to be paired with a certain symbol. The task requires completion of the gaps with the appropriate symbols in a time limit.  -It assesses speed and visuomotor ability, the handling of pencil and paper and, particularly, the capacity for associative learning. | PSI | Principal for FSIQ |
| Symbol Search (SS) | -In this task, two symbols are presented, and the participant must decide whether any symbols are present within a set.  -It evaluates perceptual speed and accuracy along with the speed with which simple visual information is processed. | PSI | Principal for PSI |

*Note.* Score range for all subtests: 1-19 (M= 10; SD= 3). FSIQ = Full-Scale IQ. VCI= Verbal Comprehension Index; VSI= Visual-Spatial Index; FRI= Fluid Reasoning Index; WMI= Working Memory Index; PSI= Processing Speed Index.

**Supplementary Table 2.**

*Reliability coefficients of WISC-V subtests of the study sample.*

|  | BD | SI | MR | DS | CD | VC | FW | VP | PS | SS |
| --- | --- | --- | --- | --- | --- | --- | --- | --- | --- | --- |
| *r_xx_* | 0.75 | 0.86 | 0.85 | 0.92 | 0.80 | 0.80 | 0.90 | 0.90 | 0.85 | 0.81 |

*Note.* Internal consistency reliabilities were directly estimated from the study sample using the Spearman-Brown formula with the exception of time limited subtests (Symbol Search and Coding) for which test-retest reliabilities from the standardization sample were used (Wechsler, 2015). BD = Block Design; SI = Similarities; MR = Matrix Reasoning; DS = Digit Span; CD = Coding; VC = Vocabulary; FW = Figure Weights; VP = Visual Puzzles; PS= Picture Span; SS = Symbol Search.

**Supplementary Table 3**

*Checking of the assumptions of the Linear regressions*

|  | Durbin Watson | VIF | Tolerance | Max. Cook’s distance |
| --- | --- | --- | --- | --- |
| **Regression 1**  Similarities  Vocabulary | 2.008 | 1.450  1.450 | 0.690  0.690 | 4.87 (there is only one case with a value higher than 1) |
| **Regression 2**  Block Design  Visual Puzzles | 1.792 | 1.660  1.660 | 0.602  0.602 | 0.44 |
| **Regression 3**  Matrix Reasoning  Figure Weights | 1.770 | 1.243  1.243 | 0.805  0.805 | 1.57 (there are two cases with a value higher than 1) |
| **Regression 4**  Digit Span  Picture Span | 1.915 | 1.392  1.392 | 0.718  0.718 | 0.18 |
| **Regression 5**  Symbol Search  Coding | 1.887 | 1.791  1.791 | 0.558  0.558 | 0.66 |
| **Regression 6**  Vocabulary  Matrix Reasoning  Picture Span  Symbol Search | 2.035 | 1.340  1.562  1.392  1.706 | 0.746  0.640  0.718  0.586 | 0.10 |
| **Regression 7**  Matrix Reasoning  Digit Span  Coding | 1.671 | 1.466  1.819  1.698 | 0.682  0.550  0.589 | 0.21 |

When checking the assumptions of the several linear regressions conducted in this study, first, the linearity assumption was tested with several scatterplots. It was also verified that no curvilinear relationship existed between our predictor variables and the dependent variable in each case.

The homocedasticity assumption was then checked with the plot of standardized residuals versus predicted values in each regression. Those plots showed no obvious signs of funneling, suggesting the assumption of homoscedasticity has been met.

Regarding the normality of the residuals, it is worth noting that in some of the regressions (in regressions 3, 4 and 5) the P-P plot for the model suggested that the assumption of normality of the residuals may have been violated. However, as only extreme deviations from normality are likely to have a significant impact on the findings, the authors consider that the results remain valid.

Regarding multicollinearity, as shown in the table (Supplementary Table 3), analysis of collinearity statistics indicates that this assumption has been met, as VIF scores were well below 10, and tolerance scores above 0.2. Thus, the authors conclude there is no multicollinearity in our data.

Regarding auto-correlation, as seen in the table, Durbin-Watson values are between 1.5 and 2.5, indicating that there is no auto-correlation in the data. Thus, the authors conclude that the values of the residuals are independent in the data set.

Finally, we checked whether any influential cases were biasing our models. In regressions 2, 4, 5, 6 and 7 Cook’s Distance values were all under 1, suggesting individual cases were not unduly influencing the model. However, in regressions 1 and 3, in one and two cases respectively, this value was higher than 1. In both cases, the analyses were rerun excluding those cases, and the results did not differ (the subtests with the highest standardized β coefficient in each regression analysis remained the same).

**Supplementary Table 4**

*Linear regression analyses for each of the five indexes of Proposal A*

| Variable | | B | *SE* | *β* | *t* | *p* |
| --- | --- | --- | --- | --- | --- | --- |
| **Regression 1** | |  |  |  |  |  |
| (Constant) | | 37.66 | 1.82 |  | 20.65 | < 0.001 |
| Similarities | | 3.00 | 0.22 | 0.516 | 13.72 | < 0.001 |
| Vocabulary | | 3.30 | 0.22 | 0.571 | 15.18 | < 0.001 |
| **Regression 2** |  |  |  |  |  |  |
| (Constant) | | 44.51 | 0.19 |  | 239.45 | < 0.001 |
| Block Design | | 2.76 | 0.02 | 0.569 | 131.39 | < 0.001 |
| Visual Puzzles | | 2.78 | 0.02 | 0.543 | 125.31 | < 0.001 |
| **Regression 3** |  |  |  |  |  |  |
| (Constant) | | 41.63 | 0.22 |  | 191.19 | < 0.001 |
| Matrix Reasoning | | 2.95 | 0.02 | 0.642 | 148.85 | < 0.001 |
| Figure Weights | | 2.88 | 0.02 | 0.533 | 123.45 | < 0.001 |
| **Regression 4** |  |  |  |  |  |  |
| (Constant) | | 42.43 | 0.35 |  | 119.89 | < 0.001 |
| Digit Span | | 2.82 | 0.05 | 0.503 | 60.85 | < 0.001 |
| Picture Span | | 2.92 | 0.04 | 0.636 | 76.91 | < 0.001 |
| **Regression 5** |  |  |  |  |  |  |
| (Constant) | | 42.36 | 0.30 |  | 142.63 | < 0.001 |
| Symbol Search | | 2.91 | 0.04 | 0.550 | 75.79 | < 0.001 |
| Coding | | 2.87 | 0.04 | 0.544 | 74.95 | < 0.001 |

*Note.* SE = Standard Error.
